# Supplementary material for: Quantum-like architecture of the social atom: mental marker coherence and the triple-resonance mechanism of cognitive activation
Source: Front Hum Neurosci. 2026 Aug 6;20:1882642. doi: 10.3389/fnhum.2026.1882642 (PMC13490188; doi:10.3389/fnhum.2026.1882642)
Supplement: Supplementary file 1 [file Data_Sheet_1.pdf]

Quantum-like Architecture of the Social Atom: Mental Marker Coherence and the Triple-Resonance Mechanism of Cognitive Activation

Andrei Khrennikov[1,*], Christian Aspalter[2], Likan Zhan[3]

Appendix: Foundational Discussion

Quantum theory offers a powerful and remarkably general mathematical framework for the rigorous analysis of quantum phenomena. However, as Niels Bohr emphasized early on, the theory does not describe the intrinsic behavior of quantum systems. Instead, it models experimental contexts and predicts, with extraordinary precision, the probability distributions of measurement outcomes.

According to the Copenhagen interpretation, as formulated by Bohr, quantum theory concerns itself with preparation and measurement procedures, and with the probabilistic relationship between them. Not all physicists accept this perspective, however. Quantum theory is characterized by a vast diversity of interpretations - even the Copenhagen interpretation itself has numerous "sub-interpretations" (and Plotnitsky has suggested referring instead to interpretations in the spirit of Copenhagen [94]–[96]). Engaging in debates over interpretation often opens a philosophical Pandora's box, and so we shall not pursue that path further here.

Our aim is simply to underline a widely held view: that quantum theory is not intended to "explain" phenomena in a classical sense, but rather to provide a calculational tool. The sentiment often attributed to Feynman - "Shut up and calculate!" - aptly captures this pragmatic stance, especially in response to demands for deeper explanations of core postulates such as Born's rule for computing quantum probabilities.

Some in the quantum community have nonetheless sought such explanations, particularly through the development of hidden variable theories. Using the Copenhagen interpretation in Bohr's spirit [94]–[96] does not preclude the possibility of constructing such theories - and we will return to this issue later. But it is important to note that introducing hidden variables goes beyond conventional quantum theory. Typically, hidden variable theories are more complex than standard quantum mechanics, which is remarkably elegant and tractable, largely due to its linear mathematical structure.

Our purpose here is to advocate for the use of conventional quantum theory beyond its traditional domain of physics - especially in the social sciences - through the further development of SLT. Still, foundational issues remain central to our approach in QLM,

because the systems under consideration are macroscopic and can, in principle, be described classically. In that sense, one might say that in QLM, hidden variables do exist.

Similarly to quantum modeling of physical processes, QLM provides a powerful and remarkably general apparatus for rigorous mathematical analysis of cognitive, sociopolitical phenomena (see above citations). Yet, QLM falls short in offering detailed explanations of these processes in terms that resonate with cognition and sociopolitical sciences. In this regard, the situation mirrors that of quantum physics.

In philosophic terms quantum theory is an epistemic theory - one that describes what can be known - formulated in terms of a peculiar and highly restricted class of observables. It is, of course, possible (though by no means necessary) to pursue ontic, or realist, theories that lie beneath quantum formalism - so-called hidden variables models. In the mainstream discourse of physics, it is often asserted - under assumptions considered "natural" - that such ontic theories are either nonexistent or physically implausible. Those that do exist are frequently dismissed as too exotic, even unphysical: Bohmian mechanics, with its nonlocality, or Nelson's stochastic mechanics, with its diffusion foundations, are prime examples. However, these sweeping no-go conclusions stem from an overly rigid and narrow framework governing the relationship between ontic and epistemic levels of theory - a framework primarily shaped by von Neumann, Bell, and Kochen-Specker.

An alternative, more generous framework is offered by *the Bild conception of theory* – after Hertz, Boltzmann, Schrödinger (see [81] for review and reinterpretation) and more recently, Atmanspacher and Primas [17]). The Bild framework loosens the straitjacket traditionally imposed on the prequantum→quantum correspondence. Within this paradigm, one may construct hidden-variable models that underpin quantum theory without the naive identification of quantum observables with prequantum variables, as Bell once attempted. A key insight of the Bild approach is that the mapping from prequantum to quantum states is not one-to-one. Many distinct prequantum configurations may project onto the same quantum state - compare, for instance, with't Hooft's views on deterministic substrata [51]. From this perspective, quantum theory offers not a mirror of reality but a blurred, approximate image - an echo rather than a voice.

That is why it is so important to also develop sociological and economic quantum theories from ground up. They are not limited by physical (or physically possible/technologically possible) boundaries. Evidence in social and economic sciences is there, it is plentiful everywhere. The problem is the opposite as in quantum physics. in social science, we have too much data, and too many variables, and this makes social science, including economics, so difficult (for those who perceive it as a science, as any other). In physics terminology, in the social sciences the degrees of freedom are often too numerous. But, not so in the case of Social Laser Theory, as it applies to special mass-scale social-psychological and political situations that (on purpose) lose details. People are losing their individuality, scores of individual traits, in social lasing events. That is, in Social Laser events only a few variables are guiding and triggering the social lasing process, effervescent mass reactions.

We are indeed in need of many more social quantum theories. As with SLT, it is common in social sciences to work on a theory for decades. Some theories get defeated and others succeed in a competitive manner, where the all-deciding difference between defeat and success is always empirical evidence, and practicability in terms of applications and their outcomes.

In SQFT building, including quantum economics theory building, theorists are not bound

by traditional or widespread believes (as they are common also in physics). Quantum social science is more flexible, as we use quantum principles and quantum physical ontology is not the only base and are not the only building blocks to work with. We must also use all practical sociological, psychological and economic theories, and then integrate quantum principles or wisdom, quantum formalism and theory wherever it brings an advantage, wherever it leads to breakthroughs and innovation.

In social quantum theory building, human entanglements are replacing quantum-level entanglements. Weak time symmetry in the quantum realm is translated into echoes of the human past, and projections of expectations and evaluations of the future. Stories, emotions, logic and experiences of the past and stories/expectations of the future both become manifestations of social realities that are also alive and causally active in the here and now [13].

In SQFT, cultural predispositions, lingual connotations, emotional and thought-to-be-logical representations all become manifestations of the past in the here and now. The past is alive and the future is alive, too, both of which are constantly and vehemently being activated, changed and re-edited in the now. Socio-cultural and socio-political environmental changes, present-day education contents, media reports, social media contents are all triggering and re-editing these manifestations, which are causal manifestations in the sense of human entanglements. There are e.g. power exertions or Foucauldian power relations of the past activated and/or amplified (or dampened) in the here and now. The activation keys of today do not contain the action imperatives planted in the past, grown and constantly re-edited from the ancient past, millennia and centuries ago, to the current day. They are emergent. Also events themselves, or news about the events themselves, can and do trigger mass responses, shifts in mass sentiments, attitudes and mass action protocols, the likelihood of all choices - of thoughts, feelings and actions [12]. SLT has focused on such mass events and mass dynamics.

The limits and paradoxes of quantum physics itself translate into quantum social science problems (e.g. the need to explain and to refer to specific and/or all interpretations of quantum physics). But additional choices in between different interpretations of quantum physics, and choices like to avoid or accept working with e.g. 'weak time symmetry' concepts all make up for difficulties that arise from a large, and growing, number of different in-between-themselves-incoherent schools (interpretations) of quantum mechanical and quantum field theories.

References

[1] Acharya, S. K., Akolina, P., & Rahman, W. (2025). Quantum Social Science: Exploring Possibility and Insights for New Genre of Social Science Research (QSS). *Journal of Community Mobilization and Sustainable Development*, 20(2), 647–659.

[2] Aerts, D. (2009). Quantum Structure in Cognition. *J. Math. Psych.*, 53(5), 314–348.

[3] Aerts, D., Arguelles, J., Beltran, L., & Sozzo, S. (2023). Development of a Thermodynamics of Human Cognition and Human Culture. *Phil. Trans. Royal Soc, A.* https://doi.org/10.1098/rsta.2022.0378

[4] Aerts, D., Gabora, L., & Sozzo, S. (2013). Concepts and Their Dynamics: A Quantum-Theoretic Modeling of Human Thought. *Topics in Cognitive Sciences*, 5, 737–772.

[5] Alodjants, A. P., Bazhenov, A. Y., Khrennikov, A. Y., & Bukhanovsky, A. V. (2022). Mean-field theory of social laser. *Scientific Reports*, 12(1), 8566.

[6] Alodjants, A., Zacharenko, P., Tsarev, D., Avdyushina, A., Nikitina, M., Khrennikov, A., & Boukhanovsky, A. (2023). Random lasers as social processes simulators. Entropy, 25(12), 1601.

[7] M. Asano, M. Ohya, Y. Tanaka, A. Khrennikov and I. Basieva, On application of Gorini–Kossakowski–Sudarshan–Lindblad equation in cognitive psychology, *OSID* **18**(1), 55–69 (2011).

[8] M. Asano, M. Ohya and A. Khrennikov, Quantum-like model for decision making process in two players game: A non-Kolmogorovian model, *Foundations of Physics* **41**(3), 538–548 (2011).

[9] Asano, M., Khrennikov, A., Ohya, M., Tanaka, Y. and Yamato, I.: Quantum Adaptivity in Biology: from Genetics to Cognition. Springer, Heidelberg-Berlin-New York (2015).

[10] Aspalter, C. (2020). *Ideal Types in Comparative Social Policy*. Routledge, Oxon.

[11] Aspalter, C. (2024). *Human Entanglement Theory: A Quantum Approach to the Study of All-Encompassing Human Communication*. Springer Nature, Singapore/Cham.

[12] Aspalter, C. (2024). Human Quantum Mechanics and Human Entanglement Theory: A New Paradigm for Social Sciences and Beyond. *Social Development Issues*, 46(2), 5.

[13] Aspalter, C. (2025). *Quantum Economics: The New Economics of Communication, Power and Power Relations*. Cheltenham: Edward Elgar.

[14] Aspalter, C. (ed.) (2026). Military Welfare versus People's Welfare - and the Role of Collective/Public Sentiments: An Introduction to Social Quantum Field Theory. In A.Y. Elveren (Ed.), *Routledge Handbook of Defense Economics*. Routledge (forthcoming).

[15] Aspalter, C. (2026). *Standard Axioms of the Forces of Human Coordination: A Quantum Economics Point of View* (forthcoming).

[16] Aspalter, C. (ed.) (2026). The Development of Social Quantum Field Theory: From Newtonian Social Science to Quantum Social Dynamics. In C. Aspalter (Ed.), *Quantum Social Science: Bridging Physics, Statistics, Psychology, Economics and Sociology* (forthcoming).

[17] Atmanspacher, H., & Primas, H. (2003). Epistemic and ontic quantum realities. In Time, quantum and information (pp. 301-321). Springer Berlin Heidelberg.

[18] Atmanspacher, h. (2004). Quantum theory and consciousness: An overview with selected examples. Discrete dynamics in Nature and Society, 2004(1), 51-73.

[19] Atmanspacher, H. Quantum Approaches to Consciousness. The Stanford Encyclopedia of Philosophy (Summer 2024 Edition), Edward N. Zalta & Uri Nodelman (eds.).

[20] Atmanspacher, H., & Römer, H. (2012). Order effects in sequential measurements of non-commuting psychological observables. Journal of Mathematical Psychology, 56(4), 274-280.

[21] Bagarello, F. (2019). *Quantum Concepts in the Social, Ecological and Biological Sciences.* Cambridge University Press: Cambridge, UK.

[22] Bagarello, F., Gargano, F., & Oliveri, F. (2023). Quantum tools for macroscopic systems. Cham, Switzerland: Springer.

[23] Bagarello, F., Gargano, F., Gorgone, M., & Oliveri, F. (2023). Spreading of information on a network: a quantum view. Entropy, 25(10), 1438.

[24] Bardella G. Franchini S. Pan L. Balzan R. Ramawat S. Brunamonti E.et al. (2024). Neural activity in quarks language: lattice field theory for a network of real neurons. Entropy 26:495.

[25] Bardella G. Franchini S. Pani P. (2024). Lattice physics approaches for neural networks. iScience 27:111390.

[26] Barthes, R. (1972). *Mythologies.* New York: Hill and Wang.

[27] Basieva, I., Cervantes, V. H., Dzhafarov, E. N., & Khrennikov, A. (2019). True contextuality beats direct influences in human decision making. Journal of Experimental Psychology: General, 148(11), 1925.

[28] Bohm, D. (1951). *Quantum Theory.* New York: Prentice-Hall.

[29] Bohm, D. (1990). A new theory of the relationship of mind and matter. *Philosophical Psychology*, 3(2), 271–286.

[30] Bohm, D. (1994). *Thought as a System.* London: Routledge.

[31] Born, M. (1954). The statistical interpretation of quantum mechanics. *Nobel Lecture Document Collection.*

[32] Benford, R. D., & Snow, D. A. (2000). Framing processes and social movements: An overview and assessment. *Annual Review of Sociology*, 26, 611–639.

[33] Bruza, P. D., Kitto, K., Ramm, B. J., and Sitbon, L. (2015). A probabilistic framework for analysing the compositionality of conceptual combinations. J. Math. Psych., 67, 26-38.

[34] Bruza, P. D., Fell, L., Hoyte, P., Dehdashti, S., Obeid, A., Gibson, A., & Moreira, C. (2023). Contextuality and context-sensitivity in probabilistic models of cognition. Cognitive Psychology, 140, 101529.

[35] Buice M. A. (2007). Field-theoretic approach to fluctuation effects in neural networks. Phys. Rev. E 75:051919.

[36] Buice M. A. (2009). Statistical mechanics of the neocortex. Prog. Biophys. Mol. Biol. 99, 53 - 86.

[37] Busemeyer, J.R.; Bruza, P.D. Quantum Models of Cognition and Decision; Cambridge University Press: Cambridge, UK, 2012; 2nd Edition, 2024.

[38] Busemeyer, J. R., & Pothos, E. M. (2013). Theoretical and Empirical Aspects of Quantum models of Cognition. Topics in Cognitive Science, 5(4), 672 - 688.

[39] Busemeyer, J. R., Wang, Z., Khrennikov, A., & Basieva, I. (2014). Applying quantum principles to psychology. *Physica Scripta*, *T163*, 014007.

[40] Busemeyer, J. R. (2023). Measurement Models in Quantum Cognition. In The Quantum-Like Revolution: A Festschrift for Andrei Khrennikov (pp. 269-279). Cham: Springer International Publishing.

[41] **Centola, D., & Macy, M. (2007). Complex contagions and the weakness of long ties. *American Journal of Sociology*, 113(3), 702-734.**

[42] Centola, D. (2018). *How Behavior Spreads: The Science of Complex Contagions*. Princeton University Press.

[43] Cervantes, V. H., & Dzhafarov, E. N. (2018). Snow queen is evil and beautiful: Experimental evidence for probabilistic contextuality in human choices. Decision, 5(3), 193.

[44] Christophe, V., & Rimé, B. (1997). Exposure to the social sharing of emotion: Emotional impact, listener responses and secondary social sharing. *European Journal of Social Psychology*, 27(1), 37–54.

[45] Cho, S., Crenshaw, K. W., & McCall, L. (2013). Toward a field of intersectionality studies: Theory, applications, and praxis. *Signs: Journal of Women in Culture and Society*, 38(4), 785–810.

[46] Conte, E., Khrennikov, A. Y., Todarello, O., Federici, A., Mendolicchio, L., & Zbilut, J. P. (2009). Mental States Follow Quantum Mechanics during perception and Cognition of Ambiguous Figures. *Open Systems & Information Dynamics*, 16, 85–100.

[47] Dafonte-Gomez, A. (2015). The key elements of viral advertising: From motivation to emotion in the most shared videos. arXiv preprint arXiv:1505.02002.

[48] Earl, J., & Kimport, K. (2011). *Digitally Enabled Social Change: Activism in the Internet Age*. MIT Press.

[49] Eberly J. H., Qian X. F., Al Qasimi A., Ali H., Alonso M. A., Gutierrez-Cuevas R. et al. (2016). Quantum and classical optics - emerging links. Phys. Scr. 91:063003.

[50] Eckler, P., & Bolls, P. (2011). Spreading the Virus: Emotional Tone of Viral Advertising and Its Effect on Forwarding Intentions and Attitudes. *Journal of Interactive Advertising*, 11(2), 1–11.

[51] Elze, H. T. (2024). Cellular automaton ontology, bits, qubits and the Dirac equation. Int. J. Quantum Inf., 22(06), 2450013.

[52] Entman, R. M. (1993). Framing: Toward Clarification of a Fractured Paradigm. *Journal of Communication*, 43(4), 51–58.

[53] Foucault, M. (1975). *Surveiller and Punir: Naissance de la Prison*. Gallimard: Paris.

[54] Freud, S. (1921). *Massenpsychologie und Ich-Analyse*. Psychoanalytischer Verlag.

[55] Gentili P. L. (2021). Establishing a new link between Fuzzy Logic, neuroscience, and quantum mechanics through bayesian probability. Molecules 26:5987.

[56] Granovetter, M. (1978). Threshold models of collective behavior. *American Journal of Sociology*, 83(6), 1420–1443.

[57] Gramsci, A. (2011). *Prison Notebooks*, Vols. 1–3. Columbia University Press: New York.

[58] Habermas, J. (1984). *The Theory of Communicative Action, Volume 1: Reason and the Rationalization of Society*. Boston: Beacon Press.

[59] Hatfield, E., Cacioppo, J. T., & Rapson, R. L. (1994). *Emotional Contagion*. Cambridge University Press.

[60] Hayek, F. (1990). *The Constitution of Liberty*. Routledge and Kegan Paul: London.

[61] Hofstadter, D. R. (2001). Analogy as the core of cognition. In D. Gentner, K. J. Holyoak, & B. N. Kokinov (Eds.), *The Analogical Mind: Perspectives from Cognitive Science* (pp. 499–538). Cambridge, MA: The MIT Press.

[62] Haven, E., & Khrennikov, A. (2013). *Quantum Social Science*. Cambridge: Cambridge University Press.

[63] Hwang, C.-L., & Yoon, K. (1981). *Multiple Attribute Decision Making: Methods and Applications*. Lecture Notes in Economics and Mathematical Systems (Vol. 186). Berlin: Springer-Verlag.

[64] Holcombe, R. G. (2018). *Political Capitalism: How Economic and Political Power Is Made and Maintained*. Cambridge: Cambridge University Press.

[65] **Jaksland, R. (2023). Why quantum mechanics is irrelevant for exploring social science knowledge. *European Journal for Philosophy of Science*, 13(3), 34.**

[66] Jasper, J. M. (2011). Emotions and social movements: Twenty years of theory and research. *Annual Review of Sociology*, 37, 285–303.

[67] Kuhn, T. S. (1970) [1962]. *The Structure of Scientific Revolutions*. Chicago: University of Chicago Press.

[68] Khrennikov, A. Yu. *Interpretations of Probability*. VSP Int. Sc. Publishers (1999); De Gruyter: Berlin (2009).

[69] Khrennikov, A. (2004). *Information Dynamics in Cognitive, Psychological, Social, and Anomalous Phenomena*. Series: Fundamental Theories of Physics. Kluwer, Dordrecht.

[70] Khrennikov, A. (2010). *Ubiquitous Quantum Structure: From Psychology to Finances*. Springer: Berlin/Heidelberg.

[71] Khrennikov, A. (2014). *Beyond Quantum*. Singapore: Jenny Stanford Publishing

[72] Khrennikov, A. (2015). Towards information lasers. *Entropy*, 17(10), 6969-6994.

[73] Khrennikov, A. (2016). 'Social Laser': action amplification by stimulated emission of social energy. *Phil. Trans. Royal Soc, A*, 374(2058), 20150094.

[74] Khrennikov, A., Toffano, Z., & Dubois, F. (2019). Concept of information laser: from quantum theory to behavioural dynamics. *The European Physical Journal Special Topics*, 227, 2133-2153.

[75] Khrennikov, A. (2020). *Social laser: application of quantum information and field theories to modeling of social processes.* Jenny Stanford Publ., Singapore.

[76] Khrennikov, A. (2021). Quantum-like model for unconscious - conscious interaction and emotional coloring of perceptions and other conscious experiences. Biosystems, 208, 104471.

[77] Khrennikov, A. (2023). *Open Quantum Systems in Biology, Cognitive and Social Sciences*. Springer.

[78] Khrennikov, A., & Yamada, M. (2025). Quantum-like representation of neuronal networks' activity: Modeling -"mental entanglement". *Frontiers in Human Neuroscience*, 19.

[79] Khrennikov, A., Iriki, A., & Basieva, I. (2025). Constructing a bridge between functioning of oscillatory neuronal networks and quantum-like cognition along with quantum-inspired computation and AI. *BioSystems*, 257, 105573.

[80] Khrennikov, A., Ozawa, M., Benninger, F., & Shor, O. (2025). Coupling quantum-like cognition with the neuronal networks within generalized probability theory. *Journal of Mathematical Psychology*, 125, 102923.

[81] Khrennikov, A. (2025). Contextual Reinterpretation of Quantum theory. Cambridge University Press, Cambridge.

[82] Khrennikov, A., Benninger, F., Oded Shor, O. Contextuality, incompatibility, and intra-system entanglement of mental markers. https://arxiv.org/abs/2603.03358

[83] Khrennikova, P. (2020). Quantum modeling of investment decisions: Contextuality and violation of the sure-thing principle. *J. Behavioral Finance*, 21(2), 170–181.

[84] Lewin, K. (1951). *Field Theory in Social Science: Selected Theoretical Papers*. Harper & Brothers.

[85] Laclau, E. (2005). *On Populist Reason*. London: Verso.

[86] Lehmann, J., Gonçalves, B., Ramasco, J. J., & Cattuto, C. (2012). Dynamical classes of collective attention in Twitter. *Proceedings of the 21st International Conference on World Wide Web*, 251–260. ACM.

[87] Luminet, O., Bouts, P., Delie, F., Manstead, A. S. R., & Rimé, B. (2000). Social sharing of emotion following exposure to a negatively valenced situation. *Cognition and Emotion*, 14(5), 661–688.

[88] McCombs, M. E., & Shaw, D. L. (1972). The Agenda-Setting Function of Mass Media. *Public Opinion Quarterly*, 36(2), 176–187.

[89] Mols, F., & Jetten, J. (2016). Explaining the appeal of populist right-wing parties in times of economic prosperity. *Political Psychology*, 37(2), 275–292.

[90] Mises, L. (1998) [1949]. *Human Action: A Treatise on Economics*. Ludwig von Mises Institute: Auburn.

[91] Orrell, D. (2024). Blinded by Science: The Empirical Case for Quantum Models in Finance. *Real-World Economics Review*, 107, 68–79.

[92] Ozawa, M., & Khrennikov, A. (2020). Application of theory of quantum instruments to psychology: Combination of question order effect with response replicability effect. *Entropy*, 22(1), 37.

[93] Ozawa M., & Khrennikov A. (2021). Modeling combination of question order effect, response replicability effect, and QQ-equality with quantum instruments. *Journal of Mathematical Psychology*, 100, 102491.

[94] A. Plotnitsky, *Epistemology and Probability: Bohr, Heisenberg, Schröder, and the Nature of Quantum-Theoretical Thinking*, Springer, Dordrecht, 2009.

[95] A. Plotnitsky, *Niels Bohr and Complementarity: An Introduction*, Springer, New York, 2012.

[96] A. Plotnitsky, What Is, Fundamentally, Quantum Mechanics? Quantum Theory as a Theory of Probability. *Int. J. Theor. Phys.*, 54, 4333–4357, 2015.

[97] Plotnitsky, A. (2023). The agency of observation not to be neglected: complementarity, causality and the arrow of events in quantum and quantum-like theories. Phil. Trans. Royal Soc. A, 381(2256), 20220295.

[98] Pribram, K. H. (1977). Holonomy and structure in the organization of perception. In J. M. Nicholas (Ed.), *Images, Perception, and Knowledge.* The University of Western Ontario Series in Philosophy of Science (Vol. 8). Dordrecht: Springer.

[99] A. Plotnitsky and E. Haven, The Quantum-Like Revolution. A Festschrift for Andrei Khrennikov with a foreword by 2022 Nobel Laureate Anton Zeilinger. Springer Nature, Berlin, 2023.

[100] Plato (1990). *Plato's Parmenides*. Ed. C. C. Meinwald, Oxford University Press.

[101] Pothos, E. M., & Busemeyer, J. R. (2009). A quantum probability explanation for violations of "rational" decision theory. *Proc. Royal Society B*, 276(1665), 2171–2178.

[102] Pothos, E., & Busemeyer, J. (2022). Quantum Cognition. *Annual Review of Psychology*, 73, 749–778.

[103] Pothos, E. M., Lewandowsky, S., Basieva, I., Barque-Duran, A., Tapper, K., & Khrennikov, A. (2021). Information overload for (bounded) rational agents. Proceedings of the Royal Society B: Biological Sciences, 288(1944), 20202957.

[104] Qadir, A. (1978). Quantum Economics. *Pakistan Economic and Social Review*, 16(3/4), 117–126.

[105] Rimé, B. (2009). Emotion elicits the social sharing of emotion: Theory and empirical review. *Emotion Review*, 1(1), 60–85.

[106] Rimé, B., Mesquita, B., Philippot, P., & Boca, S. (1991). Social sharing of emotion: New evidence and meta-analysis. In W. Stroebe & M. Hewstone (Eds.), *European Review of Social Psychology*, 2, 145–189. Wiley.

[107] Rachmad, Y. E. (2025). Influence Unbound: The Viral and Gimmick Impact on Behavior Transformation. United Nations Economic and Social Council.

[108] Rachmad, Y. E. (2025). Influence Unleashed: The Viral and Gimmick Effect on Behavior Transformation. United Nations Economic and Social Council.

[109] Schelling, T. C. (1971). Dynamic Models of Segregation. *Journal of Mathematical Sociology*, 1(2), 143–186.

[110] Gärdenfors, P. (2000). *Conceptual Spaces: The Geometry of Thought.* MIT Press.

[111] Snow, D. A., & Benford, R. D. (1988). Ideology, frame resonance, and participant mobilization. In B. Klandermans, H. Kriesi, & S. Tarrow (Eds.), *International Social Movement Research*, 1, 197–217. JAI Press.

[112] Tarrow, S. (1998). *Power in Movement: Social Movements and Contentious Politics.* Cambridge: Cambridge University Press.

[113] Tsarev, D., Trofimova, A., Alodjants, A., & Khrennikov, A. (2019). Phase transitions, collective emotions and decision-making problem in heterogeneous social systems. *Scientific reports*, 9(1), 18039.

[114] Tull, S., & Ozawa, M. (2026). Quantum-like Cognition in Process Theories: An Analysis. *arXiv preprint arXiv:2604.08604.*

[115] Wang, G., Li, J., Sun, Y., Chen, X., Liu, C., Wu, Y., Lu, M., Song, S., & Abbasi-Yadkori, Y. (2025). Hierarchical Reasoning Model. *arXiv preprint arXiv:2506.21734.*

[116] Watts, d. J. (2002). A Simple Model of Global Cascades on Random Networks. *Proceedings of the National Academy of Sciences*, 99(9), 5766–5771.

[117] Weber, M. (2012). The "Objectivity" of Knowledge in Social Science and Social Policy. In H. H. Bruun & S. Whimster (Eds.), *Max Weber*. Routledge.

[118] Wieser, F. (1926). *Das Gesetz der Macht*. Springer: Vienna.

[119] Wu, S., Hofman, J. M., Mason, W. A., & Watts, D. J. (2011). Who says what to whom on Twitter. *Proceedings of the 20th International Conference on World Wide Web*, 705–714. ACM.

[120] Zech, E., & Rimé, B. (2005). Is talking about an emotional experience helpful? Effects on emotional recovery and perceived benefits. *Clinical Psychology & Psychotherapy*, 12(4), 270–287.

[121] Brady, W.J., et al., Algorithm-mediated social learning in online social networks. Trends in Cognitive Sciences, 2023. 27(10): 947–960.

[122] Chudek, M., et al., Prestige-biased cultural learning: bystander's differential attention to potential models influences children's learning. Evolution and Human Behavior, 2012. 33(1): p. 46–56.

[123] Bärtl, M., YouTube channels, uploads and views. Convergence: The International Journal of Research into New Media Technologies, 2018. 24(1): p. 16–32.

[124] Nowak, M.A., Five rules for the evolution of cooperation. Science, 2006. 314(5805): p. 1560–3.

[125] Cinelli, M., et al., The echo chamber effect on social media. Proceedings of the National Academy of Sciences of the United States of America, 2021. 118(9).

[126] Ceylan, G., I.A. Anderson, and W. Wood, Sharing of misinformation is habitual, not just lazy or biased. Proceedings of the National Academy of Sciences of the United States of America, 2023. 120(4): p. e2216614120.

[127] Kitayama, S. and C.E. Salvador, Cultural Psychology: Beyond East and West. Annual Review of Psychology, 2024. 75: p. 495–526.

[128] Talhelm, T., et al., Large-scale psychological differences within China explained by rice versus wheat agriculture. Science, 2014. 344(6184): p. 603–8.

[129] Kay, P. and W. Kempton, What Is the Sapir-Whorf Hypothesis? American Anthropologist, 1984. 86(1): p. 65–79.

[130] Lupyan, G., et al., Effects of Language on Visual Perception. Trends in Cognitive Sciences, 2020. 24(11): p. 930–944.

[131] Regier, T. and P. Kay, Language, thought, and color: Whorf was half right. Trends in Cognitive Sciences, 2009. 13(10): p. 439–446.

[132] Jackson, J.C., V.K. Choi, and M.J. Gelfand, Revenge: A Multilevel Review and Synthesis. Annual Review of Psychology, 2019. 70: p. 319–345.

[133] Rozin, P. and E.B. Royzman, Negativity Bias, Negativity Dominance, and Contagion. Personality and Social Psychology Review, 2001. 5(4): p. 296–320.

[134] Whittaker, J., et al., Recommender systems and the amplification of extremist content. Internet Policy Review, 2021. 10(2).

[135] Brady, W.J., et al., Overperception of moral outrage in online social networks inflates beliefs about intergroup hostility. Nature Human Behaviour, 2023. 7(6): p. 917–927.
